# Association between advanced glycation end products and uveitis/scleritis activity in patients with active immune-mediated ocular inflammatory diseases

**DOI:** 10.1007/s10792-024-02980-7

**Published:** 2024-02-08

**Authors:** Nutchaya Sukon, Pitipol Choopong, Usanee Tungsattayathitthan, Nattaporn Tesavibul, Wilawan Sanpan, Sutasinee Boonsopon

**Affiliations:** Department of Ophthalmology, Faculty of Medicine Siriraj Hospital, Mahidol University, Bangkok, Thailand

**Keywords:** Advanced glycation end products, AGEs, Uveitis, Scleritis, Biomarker

## Abstract

**Purpose:**

To investigate for association between skin autofluorescence (SAF) advanced glycation end products (AGEs) and uveitis/scleritis activity in systemic inflammatory disease-related active non-infectious uveitis/scleritis patients.

**Methods:**

This cross-sectional study was conducted at Siriraj Hospital during October 2019 to March 2020. AGEs were measured by SAF method in systemic immune-related disease patients with active uveitis/scleritis, and those results were compared with those of healthy age-matched controls.

**Results:**

Thirty-one active non-infectious uveitis/scleritis patients and 31 age-matched controls were enrolled. The mean age of patients was 40.0 ± 12.8 years, and most were female (55.0%). The most common associated systemic immune-related disease was Vogt–Koyanagi–Harada disease (*n* = 14). Mean SAF AGE level in the study group compared to the control group was 2.38 ± 0.66 arbitrary units (AU) versus 2.58 ± 0.56 AU, respectively (*p* = 0.20). Multivariate analysis showed decreased SAF AGE level to be significantly associated with active ocular inflammation, (odds ratio: 0.01, 95% confidence interval: 0.00004–0.81; *p* = 0.04).

**Conclusions:**

SAF AGE level was not so far found to be a reliable biomarker for indicating uveitis/scleritis activity in systemic immune-related disease patients with active ocular inflammation.

**Clinical trial registration:**

Thai Clinical Trials Registry, https://www.thaiclinicaltrials.org/. (Reg. No. TCTR20200114004, registered date 01/01/2020, beginning date of the trial 10/01/2019).

## Background

Advanced glycation end products (AGEs) are modified protein derivatives that are produced mainly via non-enzymatic condensation reactions, the Maillard reaction; and their formation is a key pathophysiologic feature of many diseases. The Maillard reaction, introduced in the early 1900’s, is a reaction between reducing sugars and amino group in proteins, lipids, and nucleic acids through a non-enzymatic sequences of reactions forming Schiff bases and Amadori products [1]. Through further glycoxidation, these Amadori products become AGEs. In addition, methylglyoxal, glyoxal, and 3-deoxyglucosone are alternative pathways that can produce AGEs [1, 2]. AGEs can be divided into fluorescent AGEs such as pentosidine, vesperlysine, and crossline and non-fluorescent AGEs, such as N-(carboxymethyl)-lysine (CML) N-(carboxyethyl)-lysine, and pyralline [1, 3]. AGEs may act as mediators of diabetic complications and other age-related conditions like Alzheimer’s disease, decreased skin elasticity, male erectile dysfunction, pulmonary fibrosis, and atherosclerosis [4]. Previous study reported association between AGEs and degenerative ocular diseases, such as age-related macular degeneration (AMD), cataract, glaucoma, and diabetic retinopathy (DR) [5]. AGEs were also found to be related to many inflammatory processes, such as blood vessel inflammation, resulting from reaction among endothelial cells, lipids, and AGEs, with consequent release of cytokines and prostaglandin [5, 6]. Dong et al. [7] found serum AGE level to be significantly higher in uveitis patients (specifically Behçet’s disease, human leukocyte antigen (HLA)-B27-associated acute anterior uveitis, sarcoidosis, and Vogt–Koyanagi–Harada (VKH) disease) compared to healthy controls. Previously, skin biopsy, serum, saliva, and urine were used to measured specific AGE level by various methods including enzyme-linked immunosorbent assay (ELISA) technique, immunohistochemistry staining, and direct fluorescent assessment technique [3, 7, 8]. A relatively new, comparable, and non-invasive method for examining AGEs via ultraviolet tissue autofluorescence was introduced [9, 10]. With caution, by using the different AGEs measurement methods, blood and urine sampling does not necessarily reflex tissue AGE level [9, 11]. Even though, skin autofluorescent (SAF) method cannot measure non-fluorescent AGEs, but we believed that this would not be an obstacle. Pentosidine, which is a fluorescent AGE and a major glycoxidative end product [12], was played an important role in inflammatory processes [13, 14]. From the studies mentioned above, we hypothesized that AGE level measured by SAF method could be a potential marker for ocular inflammation. The aim of this study was to investigate for association between SAF AGE level and uveitis/scleritis activity in systemic inflammatory disease-related active non-infectious uveitis and/or scleritis patients.

## Methods

This prospective cross-sectional study was conducted at the outpatient clinic of the Department of Ophthalmology, Faculty of Medicine Siriraj Hospital, Mahidol University, Bangkok, Thailand, during October 2019 to March 2020. All study participants were aged older than 18 years. AGEs were measured by SAF method in systemic immune-related disease patients with active uveitis/scleritis, and those results were compared with those of healthy age-matched controls. Patients with infectious ocular inflammation, diabetes, or cardiovascular disease were excluded. This study was registered in the Thai Clinical Trials Registry, https://www.thaiclinicaltrials.org/. (reg. no. TCTR20200114004, registered date 01/01/2020, beginning date of the trial 10/01/2019).

Demographic and clinical characteristics, including gender, age, underlying diseases, body weight, and blood pressure, were collected and recorded. Hemoglobin A_1c_ (HbA_1c_) and serum creatinine (Cr) within 1 year were recorded. In order to be eligible for inclusion, study group participants must have had systemic inflammatory disease-related active uveitis and/or scleritis in at least one eye, and the type of systemic inflammatory disease was recorded. Isolated ocular inflammatory diseases, and idiopathic and infectious uveitis/scleritis were excluded. Patients were examined by one of three uveitis specialists (NT, PC, or SB). Uveitis/scleritis disease activity was recorded according to the Standardization of Uveitis Nomenclature and the Standardized Grading System for Scleritis [15, 16]. We defined active uveitis/scleritis in patients who had at least 1 of the following: anterior chamber cells ≥ 1+; anterior vitreous cells ≥ 1+; vitritis (vitreous haze) ≥ grade 2; active retinal infiltration/perivascular infiltration and/or choroidal infiltration; and/or, scleral injection ≥ 1+. Thirty-one age-matched controls were recruited and enrolled. All control group participants had no known uveitis, scleritis, or systemic inflammatory diseases. One of two investigators (NS or WS) provided information to participant candidates and recruited participants into the study after they fulfilled the criteria. All participants in both groups joined the study voluntarily.

### Procedure

Diagnoptics® (Groningen, the Netherlands) is a non-invasive ultraviolet tissue autofluorescence reader that was used to measure AGEs by SAF. Measurement was performed on the volar surface of each study participant’s forearm. The mean of three readings was recorded, and the results are reported in arbitrary units (AU). This procedure was performed by NS, UT, or WS in a semi-dark room. The skin of each patient was cleaned with 70% alcohol prior to measurement.

### Statistical analysis

Descriptive statistics were used to summarize patient characteristics. Categorical data were compared using either Chi-square test or Fisher’s exact test, and those results are presented as number and percentage. Normally distributed continuous data were compared using Student’s *t*-test, and those findings are reported as mean plus/minus standard deviation. Variables with a *p* value ≤ 0.25 in univariate analysis were entered into a stepwise backward multivariate model to identify factors independently associated with uveitis/scleritis activity. A *p* value of less than 0.05 was considered as statistically significant. Scatter distribution compared AGE value between active uveitis/scleritis group and healthy controls was demonstrated by violin plots using R-programming for statistics (R Foundation for Statistical Computing, Vienna, Austria). All statistical analyses were performed using SPSS Statistics version 18.0 (SPSS, Inc., Chicago, IL, USA).

## Results

**Fig. 1:**
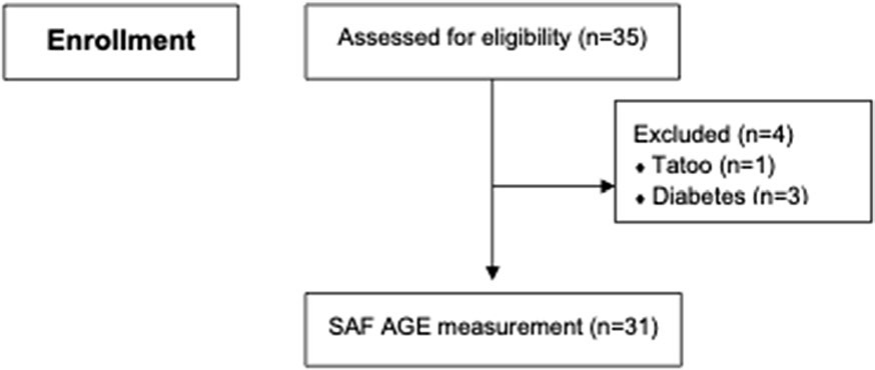
CONSORT flow diagram

**Fig. 2:**
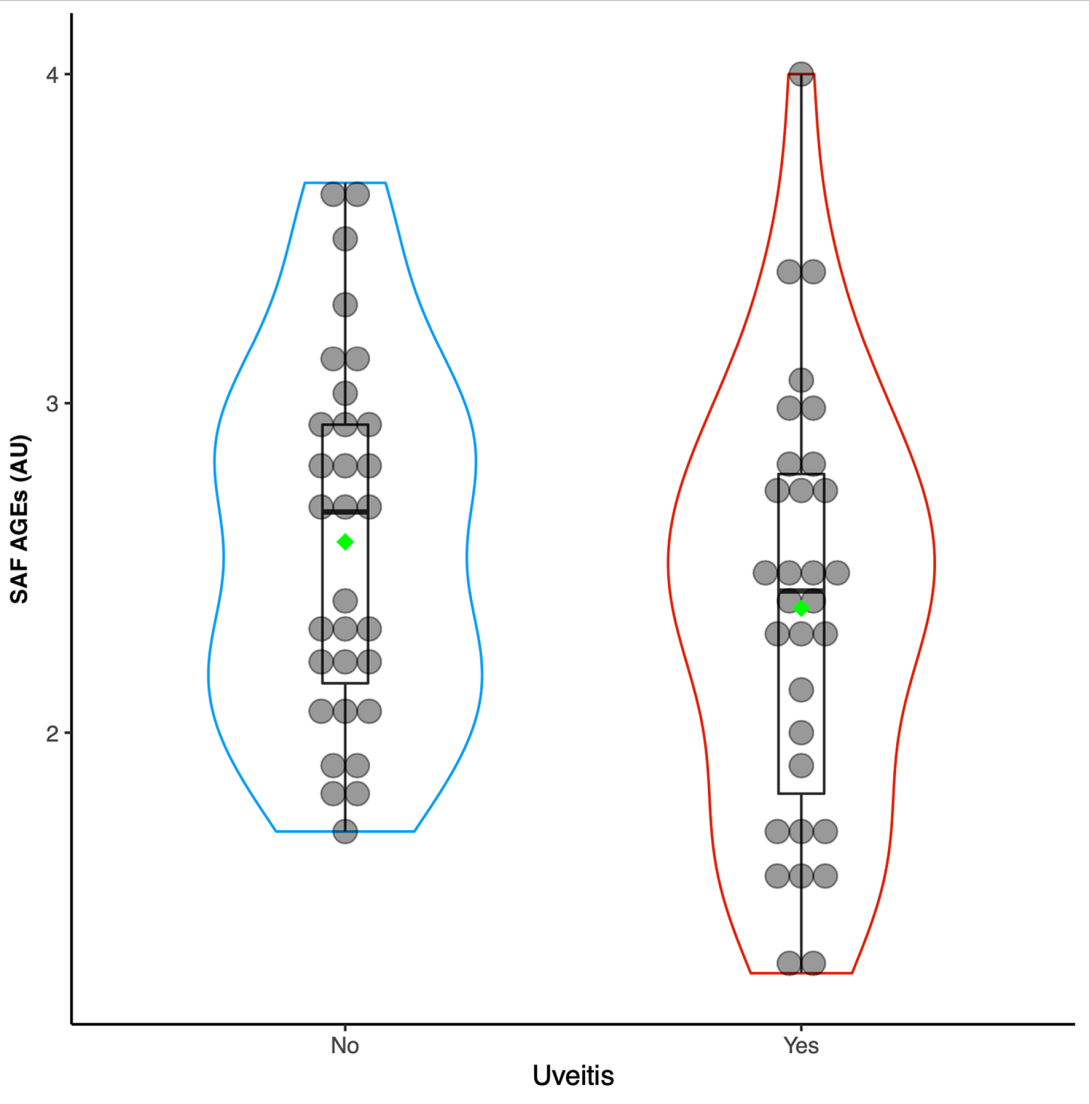
Violin plots of skin autofluorescent (SAF) advanced glycation end product (AGE) level. Violin plots demonstrate SAF AGE level distribution of patients with active uveitis/scleritis (red) and healthy controls (blue) in arbitrary unit (AU). The mean of SAF AGE level is shown in green diamond shapes and the median is shown in horizontal bold black lines

A total of 35 systemic inflammatory disease-related active non-infectious uveitis and/or scleritis patients were recruited. However, four of those patients were excluded due to 1 subject having a tattoo, and 3 subjects having diabetes. The remaining 31 study group patients were included. CONSORT flow diagram is shown in Fig. 1. The mean age of study group subjects was 40.0 ± 12.8 years (range: 22–71), and most were female (*n* = 17, 55.0%). The associated systemic immune-related diseases in the study group were Vogt–Koyanagi–Harada (VKH) disease (*n* = 14), HLA-B27-associated acute anterior uveitis (*n* = 8), Behçet’s disease (*n* = 4), sarcoidosis (*n* = 2), systemic lupus erythematosus (SLE) (*n* = 2), and rheumatoid arthritis (RA) (*n* = 1). Among HLA-B27-associated uveitis patients, six of them (75%) exhibited musculoskeletal symptoms. Specifically, three patients were diagnosed with ankylosing spondylitis, one patient experienced low back pain but was diagnosed with muscle strain, one patient had oligoarthritis with enthesitis and one patient had carpal tunnel syndrome. The vast majority of study group patients (*n* = 30, 96.8%) had active uveitis, while only 1 patient with RA presented with active scleritis. Only 4 patients were documented with active systemic disease activity. Among these 4 patients, the mean SAF AGEs level was 2.51 ± 0.3 AU (range 1.57–2.97). The most common location of intraocular inflammation was anterior uveitis (*n* = 22, 71.0%), followed by posterior uveitis (*n* = 8, 25.8%—6 without retinal vasculitis and 2 with retinal vasculitis). Most of our patients had bilateral active disease (*n* = 22, 71.0%). There were 26 patients (74.3%) who were still receiving systemic corticosteroids and/or immunomodulators or biologics as part of their treatment. Only 2 patients were on prednisolone alone, while the rests were receiving at least one immunomodulator in addition to systemic corticosteroids. The immunomodulatory therapy (IMT) and biologics included various type such as suppressor T cells like cyclosporin, antimetabolites like methotrexate, azathioprine, or mycophenolate mofetil, alkylating agents such as cyclophosphamide or chlorambucil, and tumor necrotic factor alpha inhibitors namely golimumab. Notably, 8 patients were receiving a combination of two different IMTs alongside systemic corticosteroids. In the age-match control group, there were 31 normal subjects with a mean age 40.9 ± 12.8 years (range: 22–73). The mean SAF AGE level in the study group was 2.38 ± 0.66 AU, which was non-significantly different from the 2.58 ± 0.56 AU SAF AGE level found in healthy controls (*p* = 0.2). Violin plots of SAF AGE level between active uveitis/scleritis patients and controls is shown in Fig. 2. Demographic data, clinical characteristics, and laboratory results compared between uveitis/scleritis patients and age-matched healthy controls are shown in Table 1. No variable found to be independently associated with uveitis/scleritis activity in univariate analysis. Multivariate analysis showed decreased SAF AGE level to be significantly associated with active uveitis/scleritis, (odds ratio: 0.01, 95% confidence interval 0.00004–0.81; *p* = 0.04). Univariate and multivariate analysis to identify factors independently associated with uveitis/scleritis activity is shown in Table 2. HbA_1c_ was documented in only 9 patients with active uveitis/scleritis, whereas 15 patients in the control group had performed HbA_1c_ testing. The mean of overall HbA1c was 5.49 ± 0.60.

**Table 1:** Demographic data, clinical characteristics, and laboratory results compared between uveitis/scleritis patients and age-matched healthy controls

Parameters	Uveitis/scleritis (*n* = 31)	Controls (*n* = 31)	*p*
Age (year)	40.0 ± 12.8	40.9 ± 12.8	0.77
Female gender	17 (55%)	22 (71%)	0.19
Weight (kilogram)	68.0 ± 12.8	63.5 ± 10.1	0.13
Mean arterial blood pressure (millimeter mercury)	93.4 ± 12.1	87.9 ± 12.3	0.08
Skin autofluorescence AGEs* (arbitrary unit)	2.38 ± 0.66	2.58 ± 0.56	0.2
Serum creatinine (milligram/ deciliter)	0.78 ± 0.2	0.81 ± 0.19	0.66
Hemoglobin A_1c_ (percent)	5.38 ± 0.57	5.68 ± 0.64	0.25

Data presented as mean ± standard deviation or number and percentage

A *p*-value < 0.05 indicates statistical significance

*AGEs, advance glycation end products

**Table 2:** Univariate analysis to identify factors independently associated with uveitis/scleritis activity

Parameters	Univariate			Multivariate		
	OR*	CI^†^	*p*	OR	CI	*p*
AGEs^‡^	0.57	0.24–1.34	0.2	0.01	0.00004–0.81	0.041
Female	0.50	0.17–1.42	0.19	39.03	0.85–1786.37	0.06
Age	0.99	0.95–1.03	0.76			
Weight	1.04	0.99–1.08	0.135	1.11	0.95–1.30	0.105
Mean arterial blood pressure	1.04	0.99–1.09	0.09	1.26	0.97–1.64	0.081
Serum creatinine	0.54	0.03–8.4	0.65			
Hemoglobin A_1c_	2.63	0.50–13.77	0.25	31.55	0.71–1408.44	0.075

A *p*-value < 0.05 indicates statistical significance

*OR, odds ratio; ^†^CI, confidence interval; ^**‡**^AGEs, advance glycation end products

## Discussion

The receptor for advanced glycation end products (RAGE) [17], which is responsible for amplifying inflammatory responses, had been found in a significant number in the anterior chamber, the cytoplasm of epithelial cells and stromal cells in the iris and ciliary body, vitreous cavity, ganglion cell layer, inner and outer nuclear layers, and retinal pigment epithelial cells of the retina, subretinal space, and choroid of experimental autoimmune uveoretinitis-induced rats [18]. Numbers of RAGE was found to be increased in many inflammatory diseases, which played an important role in amplifying inflammatory responses [19]. Several previous studies reported association between AGEs and inflammation [5–7, 19, 20]. In contrast, we identify inverse association between SAF AGE level and uveitis/scleritis activity in this study. Unfortunately, despite we believed that pentosidine would have reflex ocular inflammatory disease activity, but the results of this study showed negative correlation between SAF AGE level and active sclerouveal inflammation. Opposite correlation of AGE level and uveitis/scleritis activity between our study and other studies can likely be attributed to various factors. These factors may include disparity in the health status of patients and presence of underlying conditions, notably diabetes and chronic kidney disease. While we did not perform blood tests on the day of the study, we gathered retrospective data on HbA_1c_ and serum creatinine from the past year. We also believed that using diverse methods for measuring AGEs (such as serum analysis, skin biopsy, and skin autofluorescence) could potentially unveil varying levels of AGE accumulation. Finally, diet played a significant role in determining AGE levels. Influenced by distinct local cuisines and eating behavior could potentially introduce interference in the study outcomes. Corica et al. reviewed most employed techniques for detection and measurement of AGEs, along with their practical implementation within clinical settings. The most utilized AGEs measurement techniques can be categorized into 2 main groups: immunochemical methods, such as ELISA, immunohistochemistry, and Western blot; and bioanalytical method, such as fluorescence spectroscopy, SAF spectroscopy, high-performance liquid chromatography, and liquid chromatography-mass spectrometry [21]. Investigators typically consider several factors when evaluating the suitability of each technique. Advantages and disadvantages commonly assessed for each method are its simplicity, sensitivity, specificity, reproducibility, invasiveness, and cost. For SAF spectroscopy technique that we opt to use in our study is being non-invasive, rapid application, and easy to use; however, it can be interfere by other fluorophores, it cannot detect non-fluorescent AGEs, and underestimated AGE levels can be observed in individual with dark skin tone [9, 21, 22]. Kitamura et al. [23] reported a low serum glyceraldehyde derived AGE (AGE-2) subtype in active phase VKH with subsequent significantly higher AGE-2 level following high-dose corticosteroids treatment. The interpretation of these findings remained uncleared. However, the authors suggested a potential link to the immunopathogenesis of VKH disease, where melanoma-specific cytotoxic T lymphocytes played a significant role [24]. Given that AGE has the capacity to stimulate melanogenesis [25], they put forth a hypothesis that a mechanism related to AGE-2 could be involved in triggering VKH. Additionally, they postulated the existence of interactions between AGE and the RAGE in contributing to the onset of VKH disease [23]. Lastly, high incidence of glucose intolerance was found during the acute stage of VKH disease, a significant proportion of cases demonstrated an improvement in glucose intolerance subsequent to the administration of systemic corticosteroid therapy [26]. However, some studies have failed to demonstrated a consistent relationship between abnormal glucose metabolism and serum AGEs [27, 28]. The exact mechanism by which AGEs contribute to insulin resistance remains unclear [23]. On the contrary, Dong et al. found a significantly higher serum AGE level in uveitis patients compared to healthy individuals. In their study, the serum AGE levels were assessed across different conditions, including HLA-B27 related uveitis (*n* = 22), VKH disease (*n* = 20), Behçet’s disease (*n* = 24), and sarcoidosis (*n* = 44). The measure levels were 7.38 ± 0.57, 7.27 ± 1.19, 6.74 ± 0.24, and 6.45 ± 0.97 U/ml, respectively, in comparison to healthy controls at 4.16 ± 0.26 U/ml (*p* < 0.01 for Behcet’s disease and *p* < 0.001 for the other conditions). In their study, experimental mouse model of human endogenous uveitis was used to measured serum AGE level before and after pyridoxamine administration, immunohistochemistry staining, and immunologic responses They found a significant lower serum AGE levels in the group that received 400 mg/kg/day of pyridoxamine administration. On Day 10, the serum AGE levels in this group was 31.9 ± 3.9 U/ml, and on Day 21, it was 36.6 ± 5.4 U/ml. In contrast, the control group, which received deuterium-depleted water, exhibited higher serum AGE levels at 50.3 ± 7.0 U/ml on Day 10 and 56.3 ± 3.0 U/ml on Day 21. The differences were statistically significant at a *p* value < 0.05 [7]. Unfortunately, serum AGEs and skin biopsy were not done in this study. Advantages of serum and skin biopsy AGEs over SAF AGE level is its ability to identify a specific type of AGEs, and it is not interfered by skin tone. Moreover, non-fluorescent AGEs can be detected by ELISA technique when serum is used for quantitative and qualitative measurements. In the context of uveitis, we discovered a lack of studies that have identified specific subtypes of AGEs for each disease. It is possible that only some specific AGEs may relate to active ocular inflammation, but the results of this study could not reveal those outcomes. Lastly, it is important to acknowledge that the presence of IMT in the majority of our study group has the potential to influence the measurement of AGEs levels.

### Dietary concerns

Previous study proposed that a low glycemic diet would help to reduce AGE formation, and delay or diminish the development of ocular tissue damage [29]. Animal studies found a relationship between dietary AGE consumption and chronic low-grade inflammation. Dietary intervention between a high-AGE diet and a low-AGE diet revealed association between dietary intake and inflammatory markers. In a human intervention study, the investigators found evidence to support the findings of studies in animal models [20]. A mitochondria-targeted reagent that was developed by Pun et al. and a phosphate binder could reduce circulating AGEs and reduce absorption of dietary AGEs [30, 31]. Supplements, such as green tea extract, garlic, ascorbic acid (vitamin C), and thiamin (vitamin B1), were able to inhibit AGE formation [32–34]. Antioxidants, including rutin (flavonoid) and α-tocopherol (vitamin E), could also reduce AGE production [35]. Citric and acidic ingredients seemed to be able to reduce AGE formation [36]. It is possible that patients with known immune-mediated diseases may make healthy food choices by consuming a low-AGE diet which results in lower levels of AGEs in their body. This could potentially manifest as lower SAF AGE levels in our study.

## Limitations

This study has some mentionable limitations. First, even though we studied SAF in a single racial (Southeast Asian) population, skin color is still troublesome when using this technique. Moreover, skin biopsy and serum AGE levels were not investigated in this study, so we were not able to compare SAF AGE levels and AGE levels from skin biopsy and serum. Second, ocular inflammation may not represent systemic inflammation, and known inflammatory biomarkers were not recorded. Third, cross-sectional study might interfere the study results since the deposition of AGE in the skin is the time necessary for AGE to accumulate. Fourth, due to the cross-sectional nature of the study, a comparison between pre and posttreatment AGE levels could not be performed. Fifth and last, the results of our study may have been adversely influenced by the relatively small number of patients studied.

## Conclusion

SAF AGE level as a biomarker for indicating uveitis/scleritis activity in systemic immune-related disease patients with active uveitis/scleritis is inconclusive. Further investigations with larger study population can be considered to search for associations between AGEs and ocular inflammation. Confounding factors, such as skin tone, need to be adjusted. Conventional methods using serum and/ or skin biopsy may be more useful to identify specific AGEs in ocular inflammatory diseases.

## Data Availability

The datasets used and/or analyzed during the current study available from the corresponding author on reasonable request.

## Abbreviations

AGEs: Advanced glycation end products
AMD: Age-related macular degeneration
AU: Arbitrary units
CML: N-(carboxymethyl)-lysine
Cr: Creatinine
DR: Diabetic retinopathy
ELISA: Enzyme-linked immunosorbent assay
HbA_1c_: Hemoglobin A_1c_
HLA: Human leukocyte antigen
IMT: Immunomodulatory therapy
RA: Rheumatoid arthritis
SAF: Skin autofluorescence
SLE: Systemic lupus erythematosus
VKH: Vogt–Koyanagi–Harada
